# The value of white blood cell count and platelet count in predicting delayed cerebral ischemia after aneurysmal subarachnoid hemorrhage

**DOI:** 10.1016/j.bas.2025.104236

**Published:** 2025-03-13

**Authors:** Eva Postma, Homeyra Labib, Jordi van Lange, Bert Coert, René Post, René van den Berg, Charles Majoie, W. Peter Vandertop, Dagmar Verbaan

**Affiliations:** a Amsterdam UMC, Location AMC, University of Amsterdam, Department of Neurosurgery, Meibergdreef 9, Amsterdam, the Netherlands; b Amsterdam UMC, Location AMC, University of Amsterdam, Department of Radiology and Nuclear Medicine, Meibergdreef 9, Amsterdam, the Netherlands; c Amsterdam UMC, University of Amsterdam, Amsterdam Neuroscience, Neurovascular Disorders, Amsterdam, the Netherlands

**Keywords:** Aneurysmal subarachnoid hemorrhage, Delayed cerebral ischemia, White blood cell count, Platelet count, Prediction

## Abstract

**Introduction:**

Delayed cerebral ischemia (DCI) after aneurysmal subarachnoid hemorrhage (aSAH) contributes significantly to mortality and morbidity. Neuroinflammation and platelet activation are implicated in its pathophysiology.

**Research question:**

This study evaluates the association of admission white blood cell count (WBC) and platelet count (PC), and their combination, with DCI and explores their integration into predictive models.

**Materials and methods:**

This single-center cohort study utilized data from a prospective SAH registry (December 2011–December 2019). Patients with confirmed aSAH and recorded WBC and PC within 72 h post-ictus were included. Univariate and multivariate regression models with established predictors, consisting of the modified Fisher scale (mFS) and World Federation of Neurological Surgeons grade (WFNS), were performed. Predictive values were assessed using AUCs (95 % CI) and C-statistics.

**Results:**

Of 954 reviewed patients, 660 met inclusion criteria, with 178 (27.0 %) developing DCI. Patients who developed DCI had significantly higher admission WBC levels (mean (SD) 14.3 (5.1) × 10^9^/L vs. 12.7 (4.8) × 10^9^/L, p < 0.001), whereas admission PC did not differ significantly (median (IQR) 255 (201–301) × 10^9^/L vs. 241 (205–289) × 10^9^/L, p = 0.196). WBC was predictive of DCI (OR 1.06, 1.03–1.10), but PC was not (OR 1.00, 1.00–1.02). Of established predictors, mFS was significant (OR 6.42, 1.96–21.02), whereas WFNS was not (OR 0.79, 0.54–1.15). Among all variables, WBC demonstrated highest predictive value (AUC: 0.59, 0.54–0.64), surpassing mFS and WFNS, or their combination. A combined model incorporating WBC, PC, mFS, and WFNS yielded the highest predictive value (AUC: 0.63, 0.58–0.68).

**Discussion and conclusion:**

Admission WBC and PC offer modest predictive value for DCI, either alone or combined with neurological status and hemorrhage burden. However, WBC demonstrated highest predictive value of all investigated variables and modestly improves prediction models. Future research should evaluate WBC’s utility in models with enhanced predictive performance.

## Highlights

• WBC on admission is significantly associated to delayed cerebral ischemia (DCI) in aSAH patients.
• WBC predicts DCI better than the established predictors modified Fisher scale and WFNS, alone or combined.
• Incorporating WBC into models slightly improves prediction of patients at risk for DCI.
• Future studies should explore WBC in advanced models to better predict DCI.

## 1 Introduction

Aneurysmal subarachnoid hemorrhage (aSAH) is a severe type of hemorrhagic stroke and is caused by the rupture of an intracranial aneurysm. Despite advances in neurocritical care over the last few decades, aSAH remains associated with high mortality and morbidity rates (Rinkel and Algra, 2011). Case-fatality rate is reported to be 30 %, and only 36–55 % of survivors regain independence (modified Rankin Scale score 0–3) by one year after the event (Vergouwen et al., 2016; Nieuwkamp et al., 2009).

In patients who survive the initial impact of aSAH and potential rebleeding of the aneurysm, the greatest contributor to mortality and poor outcome is delayed cerebral ischemia (DCI) (Stienen et al., 2018; Ferguson and Macdonald, 2007). DCI is characterized by a significant reduction in cerebral blood flow, often due to complex interactions between vasospasm, microcirculatory disturbances, and inflammatory responses. DCI occurs in roughly one third of all patients and usually develops in 4–14 days after the ictus (Roos et al., 2000; Park et al., 2018). Clinically, it is associated with new or worsening neurological deficits, therefore diagnostic criteria for DCI include clinical deterioration coupled with imaging evidence of reduced cerebral perfusion. Diagnosing DCI can be challenging, as neurological deterioration can also result from other causes, such as hydrocephalus, infections or metabolic disruptions. Consequently, the challenge is to identify patients at risk before DCI-related symptoms occur.

Several factors are known to be associated with the development of DCI (Hijdra et al., 1988). Most frequently used in prediction models are neurological condition on hospital admission, often represented by the Word Federation Neurological Surgeons (WFNS) grade, and the amount of blood on non-contrast computed tomography (NCCT), often represented with modified Fisher Scale (mFS) (Hijdra et al., 1988; Frontera et al., 2006; de Rooij Nicolien et al., 2013; Adams et al., 1987). In addition to these established predictors, which are often subject to initial evaluation and procedures, incorporation of physiological data into a prediction model for DCI might prove useful.

It is proposed that a state of neuroinflammation, platelet activation and microthrombosis is present in patients with aSAH, and even more distinct in patients who subsequently develop DCI (Macdonald, 2014; Dodd et al., 2021). Many studies investigated a wide range of biochemical markers associated with DCI, e.g. white blood cell count (WBC), neutrophil-lymphocyte ratio (NLR), platelet-lymphocyte ratio (PLR), platelet activation, systemic inflammation response index (SIRI) and systemic immune-inflammation (SII) (Al-Mufti et al., 2019a; Tao et al., 2017; Frontera et al., 2017; Yun et al., 2021). These findings support the notion that inflammation and platelet activation are more pronounced in patients who develop DCI.

White blood cell count (WBC) and platelet count (PC) are readily accessible biomarkers that reflect distinct yet interconnected processes associated with DCI (Dodd et al., 2021). While both markers have been studied individually in relation to DCI, their potential combined effect remains less well understood. In this study, we aimed to assess the association of WBC and PC with DCI and evaluate whether their interaction offers additional predictive value in a large prospective cohort of SAH patients.

## 2 Methods

### 2.1 Study cohort

Patients admitted between December 2011 and December 2019 to the department of Neurosurgery at the Amsterdam University Medical Centre, a tertiary referral centre for the treatment of SAH patients in the Amsterdam metropolitan area with a total population of approximately 2.5 million people, were included if the following criteria were met: (1) diagnosis of SAH confirmed by CT or lumbar puncture (LP); (2) an aneurysm responsible for the SAH confirmed by CT/DS/MR-angiography; (3) age of ≥18 years; (4) WBC and PC determined with maximum time period of 72 h between ictus and first blood count; (6) a known DCI status. Patients were excluded when onset of aSAH was unknown. Furthermore, patients were excluded when death or DCI occurred within 72 h of onset.

### 2.2 Clinical management

All patients received treatment according to local standardized aSAH protocol (Connolly et al., 2012; Neurology, 2013). Patients underwent daily monitoring of physiological parameters, with hourly controls of vital signs. Neurological monitoring included repeated Glasgow Coma Scale (GCS) and pupillary reflex assessments to detect clinical deterioration. In sedated and intubated patients, neurological evaluation was performed through intermittent sedation pauses, with their frequency depending on ventricular size on initial CT imaging. If sedation pauses were not feasible, repeat CT imaging was performed to rule out hydrocephalus. Sedation was discontinued if aneurysm treatment could not be performed within 24 h to allow for clinical evaluation.

Consensus for the aneurysm treatment was attained by neurosurgeons and neuroradiologists. After aneurysm treatment a mean arterial pressure (MAP) between 65 and 135 mmHg was maintained. Standard medical treatment consisted of Nimodipine 60 mg six times daily and intravenous hydration with 0.9 % saline to maintain euvolemia, with a minimum fluid intake of 2L per day. Patients received Acetaminophen 1000 mg four times daily as starting point for analgesics, and no non-steroidal anti-inflammatory drugs (NSAIDs) were administered. To prevent thrombosis patients received prophylactic Nadroparin and wore leg stockings during hospital stay.

Currently, no proven effective treatment for DCI exists. The most investigated approach is hypertension induction therapy (HIT). While international guidelines recommend its use, Dutch guidelines suggest it should be applied only under strict indications. Intra-arterial papaverine, nimodipine or mechanical dilation (balloon angioplasty/stent) have no role in treating DCI in our Dutch or local guidelines and were therefore not initiated.

Patients with suspected or diagnosed DCI were treated with HIT in the ICU. If initiated, HIT was carried out with a target MAP and a maximum norepinephrine dose of 0.5 μg/kg/min. ECG and cardiac enzymes were monitored daily if norepinephrine exceeded 0.2 μg/kg/min or if the patients had a cardiovascular history. The target MAP was set initially at +20 mmHg above baseline, but not beyond 120 mmHg to avoid systemic complications. Patients with a spontaneous MAP >120 mmHg at DCI onset did not require treatment or ICU admission. Clinical improvement was assessed 1 h after reaching the target MAP or after dosing adjustments if the target MAP was not reached. HIT was considered effective with at least a 1-point improvement on the Glasgow Coma Scale or improvement in focal neurological deficits. If no clinical improvement occurred, norepinephrine was tapered off over 2 h, and the patients were returned to the ward. If there was clinical improvement, the target MAP was maintained for 24 h, then reduced by 10 mmHg every 12 h. If neurological symptoms worsened during tapering, the MAP was increased back to the previous effective level. Treatment was considered complete once the target MAP was successfully reduced to baseline or maintained for 12 h without norepinephrine, allowing the patient to return to the ward.

### 2.3 Data collection and variable definition

From the prospective SAH-registry in which all SAH patients admitted to the Amsterdam UMC are documented since 2011, the following demographic and clinical data were retrieved: age, sex, medical history, neurological status on admission, aneurysm treatment modality, complications during hospital stay, and DCI status. Radiological data included mFS on primary NCCT scan and aneurysm location on CT-angiography (CTA) and/or digital subtraction angiography (DSA). Neurological condition was assessed at the primary hospital by means of WFNS grade (Drake, 1988). The WFNS is an ordinal scale ranging from 1 to 5, with higher scores indicating worse neurological status, and is based on the Glasgow Coma Scale (GCS). Intubation was counted as V1 in the GCS verbal subscore.

WBC and PC at admission were retrospectively collected from electronic healthcare records. Normal ranges are 4.0–10.5 × 10^9^/L for WBC and 150–400 × 10^9^/L for PC. We maintained a strict 72 h interval from aSAH ictus to first WBC and PC determination, generally preceding DCI onset. Ictus times of aSAH were classified as exact or estimated with a margin of guess. The margin was defined in minutes, hours or days. Whenever a margin of guess existed, it was subtracted from the estimated ictus time to obtain the earliest possible ictus time.

DCI occurrence was monitored throughout hospital admission and primarily assessed through clinical evaluation. The following definition by Vergouwen et al. was maintained: “Occurrence of focal neurological impairment (…), or a decrease of at least 2 points on the Glasgow Coma Scale (…), which was not apparent immediately after aneurysm occlusion, and cannot be attributed to other causes by means of clinical assessment, imaging of the brain, and appropriate laboratory studies” (Vergouwen et al., 2010). When necessary, a NCCT- or MR-scan was performed to exclude alternative explanations such as hydrocephalus, rebleeding, or infarction.

We decided in advance to incorporate mFS (radiological variable) and WFNS grade (clinical variable) into the models, as these are well established predictors. (Hijdra et al., 1988; Frontera et al., 2006; de Rooij Nicolien et al., 2013; Adams et al., 1987). mFS was dichotomized into good grade (thin SAH, 0–2) and poor grade (thick SAH, 3–4), WFNS was dichotomized into good grade (1–3) and poor grade (4–5), due to insufficient values in certain categories and because this is commonly done in the literature.

### 2.4 Standard protocol approvals, registrations, and patients consents

Approvals for this retrospective cohort study and the prospective SAH registry were obtained from the Institutional Review Board committee (Medical Ethics Committee AMC, reference number resp. W19_297, 19.354 and W11_145, 11.17.0979). This study was conducted in accordance with the Declaration of Helsinki and institutional ethics guidelines. Data will be made available on request of any qualified investigator.

### 2.5 Statistical analysis

Descriptive data were reported as frequencies (percentage) for categorical data, as mean (standard deviation (SD)) for normally distributed continuous data, and as median (interquartile range (IQR)) for non-normally distributed data. Normality was evaluated using the Shapiro-Wilk test (with a cut-off of 0.90, where values above this threshold were considered indicative of normal distribution), alongside visual inspection via histograms and Q-Q plots. Differences between groups of patients who did or did not develop DCI were evaluated using chi-square test for dichotomous or categorical variables, Student’s *t*-test for normally distributed data, and Mann Whitney *U* test for non-normally distributed data.

Univariate logistic regression models were used for the assessment of the association of admission WBC and PC with DCI. Multivariate regression models were used to investigate their combined effect, and to incorporate predetermined predictors mFS and WFNS. Odds ratios (OR) and 95 % confidence intervals (CI) and p-values were reported. Multicollinearity between WFNS and mFisher was assessed using Variance Inflation Factor (VIF) in a linear regression model, with values above 5 considered indicative of significant collinearity. Considering that WBC and PC may both contribute to the pathophysiology underlying the development of DCI – whether independently or in an interconnected manner – we also sought to explore the potential for effect modification. To evaluate this, we fitted a model including all previously described predictors along with a multiplicative interaction term between WBC and PC, in line with recommendations from the literature (Riley et al., 2022; Altman and Bland, 2003). We fitted a model including all previously described predictors along with a multiplicative interaction term between WBC and PC. The significance of this interaction was assessed using a log-likelihood ratio test comparing models with and without the interaction term. For illustrative purposes, the conditional effects of WBC and/or PC on DCI at different levels are presented as a plot.

To assess discrimination ability of WBC and PC in predicting DCI, we estimated receiver operator characteristic curves (ROC) and concomitant areas under the curve (AUC), and the C-statistic, with corresponding 95 % CIs.

A sensitivity analysis was performed to assess the robustness of our findings. In this analysis, we excluded only patients who developed DCI prior to the first blood sample, rather than all patients with DCI or death within 72 h after ictus. This approach ensured that the measured WBC and PC reflected the pre-DCI state.

Statistical analyses were performed using IBM SPSS Statistics 26.0 and R version 4.4.1. Differences were considered significant if two-sided p < 0.05.

## 3 Results

**Fig. 1:**
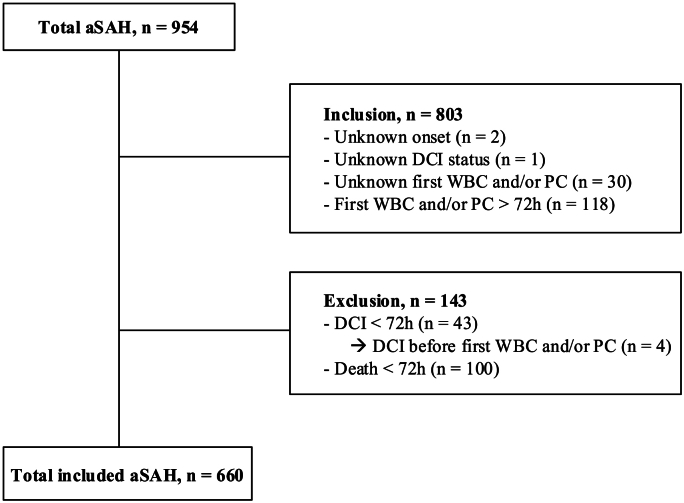
Flowchart of included and excluded patients. ‘Inclusion’ shows elements of inclusion criteria described in the method section. aSAH = aneurysmal subarachnoid hemorrhage; DCI = Delayed Cerebral Ischemia; WBC = White blood cell count, PC = Platelet count.

Of 954 aSAH patients reviewed, 660 patients met the inclusion criteria (flowchart Fig. 1). Mean (SD) age of all included patients was 57.0 (12.8) years, and 70.6 % were female. Median (IQR) WFNS score was 2 (1–4), median (IQR) mFS was 4 (3–4) (Table 1).

**Table 1:** Demographic, clinical and radiological data of all included patients; DCI and no DCI.

	Total		DCI		No DCI		P-value
No. of patients	660		178	27.0 %	482	73.0 %	
Female sex^a^	466	70.6 %	134	75.3 %	332	68.9 %	0.109
Age †	57.0	12.8	56.8	12.5	57.0	12.9	0.825

History							
Hypertension^a^	160	24.2 %	39	21.9 %	121	25.1 %	0.396
Cardiovasc. disease^a^	96	14.5 %	28	15.7 %	68	14.1 %	0.600
Diabetes Mellitus^a^	26	3.9 %	5	2.8 %	21	4.4 %	0.364

Admission							
WFNS^a^							0.615
I	267	40.5 %	66	37.1 %	202	41.9 %	
II	128	19.4 %	38	21.3 %	89	18.5 %	
III	26	3.9 %	8	4.5 %	19	3.9 %	
IV	144	21.8 %	36	20.2 %	107	22.2 %	
V	95	14.4 %	30	16.9 %	65	13.5 %	
mFS^a^							0.003
0	14	2.1 %	–	–	14	2.9 %	
1	25	3.8 %	2	1.1 %	23	4.8 %	
2	15	2.3 %	1	0.6 %	14	2.9 %	
3	163	24.7 %	40	22.5 %	123	25.5 %	
4	443	67.1 %	135	75.8 %	308	63.9 %	

Aneurysm							
Location^a^							0.303
Anterior circulation	405	62.4 %	108	60.7 %	297	61.6 %	
Posterior circulation	243	36.8 %	69	38.8 %	174	36.1 %	
Other	12	1.8 %	1	0.6 %	11	2.3 %	
Treatment^a^							0.003
Coiling	492	74.5 %	132	74.2 %	360	74.7 %	
Clipping	105	15.9 %	39	21.9 %	66	13.7 %	
No	47	7.1 %	4	2.2 %	43	8.9 %	
Other	16	2.6 %	3	1.7 %	13	2.7 %	

Complications^a^							
Hydrocephalus	435	65.9 %	144	80.9 %	291	60.4 %	<0.001
Rebleed	113	17.1 %	36	20.2 %	77	16.0 %	0.198
Fever	267	40.5 %	101	56.7 %	166	34.4 %	<0.001
Meningitis	45	6.8 %	23	12.9 %	22	4.6 %	<0.001
Pneumonia	103	15.6 %	35	19.7 %	68	14.1 %	0.081
UTI	55	8.3 %	18	10.1 %	37	7.7 %	0.315
Sepsis	13	2.0 %	8	4.5 %	5	1.0 %	0.005
DVT	1	0.2 %	–	–	1	0.2 %	0.543
Delirium	117	17.7 %	49	27.5 %	68	14.1 %	<0.001
Epilepsy	114	17.3 %	50	28.1 %	64	13.3 %	<0.001

Outcome at 6 months– modified Rankin Scale							0.001
0 – no symptoms	29	4.4 %	28	5.8 %	1	0.6 %	
1 – non-significant	54	8.2 %	46	9.5 %	8	4.5 %	
2 – slight	251	38 %	188	39 %	63	35.4 %	
3 – moderate	68	10.3 %	47	9.8 %	21	11.8 %	
4 – moderate/severe	34	5.2 %	19	3.9 %	15	8.4 %	
5 – severe	44	6.7 %	29	6 %	15	8.4 %	
6 – dead	130	19.7 %	86	17.8 %	44	24.7 %	
Missing	50	7.6 %	39	8.1 %	11	6.2 %	

a n (%); † Mean ± SD. DCI = Delayed Cerebral Ischemia; mFS = modified Fisher Scale; SD = standard deviation; WFNS = World Federation of Neurosurgical Societies grade; UTI = urinary tract infection; DVT = deep vein thrombosis.

A total of 178 patients (27.0 %) developed DCI. First WBC after ictus was determined at a median (IQR) of 4.0 h (2.0–10.0), and first PC at a median (IQR) of 4.0 h (2.0–9.0). In the whole cohort of aSAH patients the mean WBC was above normal range (13.2 (5,0) × 10^9^/L), the median PC was within normal range (245 (204–291) × 10^9^/L).

At 6 months, DCI patients had significantly worse functional outcomes compared to non-DCI patients (p < 0.001), with a shift toward higher modified Rankin Scale (mRS) scores (4–6).

VIF values for WFNS and mFisher were both 1.107, indicating no significant collinearity between these variables.

### 3.1 Relation between WBC, PC and DCI

**Fig. 2:**
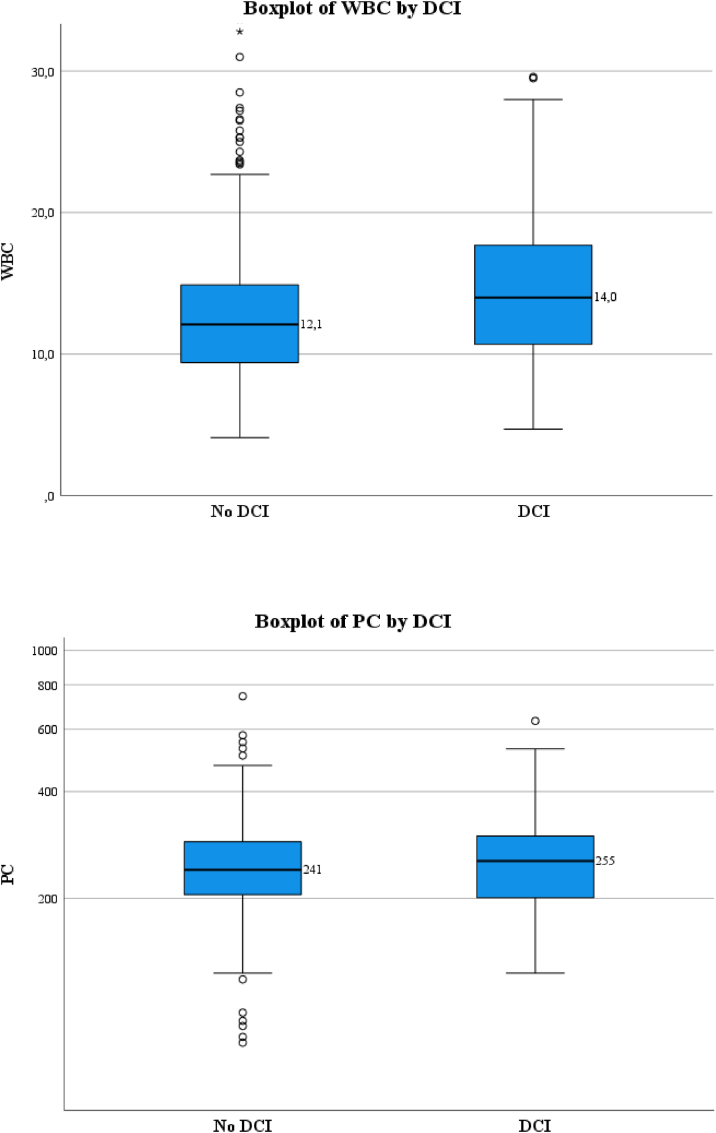
Boxplots of WBC and PC by occurrence of DCI, with median (thick black line) and interquartile range (whiskers) DCI = delayed cerebral ischemia; WBC = White blood cell count; PC = Platelet count. Note the linear scale in the first and the logarithmic scale in the second boxplot.

Patients who developed DCI had higher admission WBC than patients without DCI (mean (SD) resp. 14.3 (5.1) vs. 12.7 (4.8) × 109/L, p < 0.001), admission PC did not differ between groups (median (IQR) resp. 255 (201–301) × 109/L vs 241 (205–289) × 109/L, p = 0.196). Fig. 2 displays boxplots of the distribution of WBC and PC by occurrence of DCI. WBC is associated with the occurrence of DCI (OR 1.07, CI 1.03–1.10), PC alone was not associated with DCI (OR 1.00, CI 0.99–1.00). Their combination showed ORs of resp. 1.07 (CI 1.03–1.10) and 1.00 (CI 1.00–1.00). Table 2 shows results of the uni- and multivariate regression analyses.

**Table 2:** Odds ratios for the model with all variables.

	OR	95 % CI	P-value
***Univariate models***			
WBC	1.07	1.03–1.10	<0.001
PC	1.00	0.99–1.00	0.449
***Multivariate models***			
**Qualifying variables combined**			
WBC	1.07	1.03–1.10	<0.001
PC	1.00	1.00–1.00	0.918
**Predictors combined**			
mFS	7.06	2.16–23.03	<0.001
WFNS	0.93	0.65–11.34	0.702
**Qualifying variables combined with predictors**			
WBC	1.06	1.03–1.105	<0.001
PC	1.00	1.00–1.002	0.895
mFS	6.42	1.96–21.02	0.002
WFNS	0.79	0.54–1.15	0.220

DCI = delayed cerebral ischemia; WBC = white blood cell count, PC = platelet count; mFS = modified Fisher Scale (0–2 vs. 3–4); WFNS = World Federation of Neurosurgical Societies grade (1–3 vs. 4–5). OR = odds ratio; 95 % CI = 95 % confidence interval. Note that both mFS and WFNS are dichotomized.

Given that WBC showed a significant association with DCI, we subsequently fitted a regression model including a multiplicative interaction term between WBC and PC to assess potential effect modification. The interaction was not statistically significant as confirmed by a log-likelihood ratio test comparing the regression model including the interaction term to a model without the interaction term (p = 0.43). A marginal plot of the predicted probabilities (available in the supplementary material) further illustrates that variations in PC do not meaningfully alter the association between WBC and the risk of DCI.

### 3.2 Discrimination and predictive accuracy of the variables and models

**Fig. 3:**
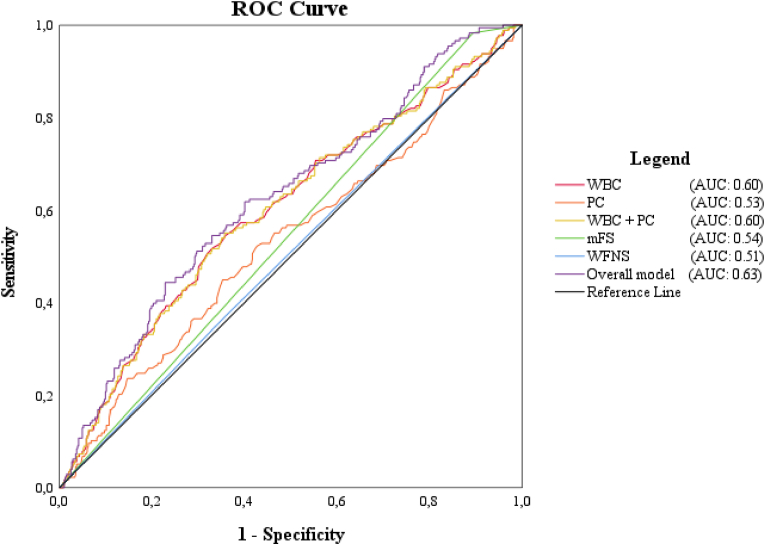
ROC Curve and AUC of variables WBC, PC, mFS, WFNS and their combined model ROC Curve = Receiver under the Operator Characteristic Curve; AUC = area under the curve; WBC = white blood cell count; PC = platelet count. Modified Fisher scale (0–2 vs. 3–4); WFNS = World Federation of Neurosurgical Societies grade (1–3 vs. 4–5).

ROC curve analysis revealed that WBC (AUC: 0.60, CI 0.55–0.65) had better predictive abilities for DCI than both established predictors mFS (AUC: 0.54, CI 0.50–0.59) and WFNS (AUC: 0.51, CI 0.46–0.56) or their combination (AUC: 0.55 (CI 0.50–0.65) (Table 3). Fig. 3 shows the ROC-curve of separate WBC, PC, mFS, WFNS; furthermore, the combination of WBC and PC, and the model combining all variables. The model combining all variables yielded moderate, yet highest, predictive value of all evaluated variables, with an AUC of 0.63 (CI 0.58–0.67). The C-statistic showed a discrimination of the model of 0.62 (CI 0.57–0.67).

**Table 3:** Area under the curve of all variables and model.

	*AUC*	*95 % CI*
**Univariate models**		
*WBC*	0.60	0.55–0.65
*PC*	0.53	0.48–0.58
*mFS*	0.54	0.50–0.59
*WFNS*	0.51	0.46–0.56
**Multivariate models**		
*WBC + PC*	0.60	0.55–0.65
*mFS + WFNS*	0.55	0.50–0.60
*WBC + PC + mFS + WFNS*	0.63	0.58–0.67

DCI = delayed cerebral ischemia; PC = platelet count; WBC = white blood cell count, mFS = modified Fisher Scale (0–2 vs. 3–4); WFNS = World Federation of Neurosurgical Societies grade (1–3 vs. 4–5). AUC = area under the curve; 95 % CI = 95 % confidence interval.

### 3.3 Sensitivity analysis

Four patients developed DCI before first WBC and PC, so in the sensitivity analysis the cohort consisted of 799 patients. The results remained similar with the mean (SD) WBC remained significantly higher in patients who developed DCI compared with those who did not (14.4 (5.7) vs. 13.0 (5.1) × 10^9^/L, p = 0.001), while median (IQR) PC did not differ significantly (248 (203–294) vs. 241 (202–287) × 10^9^/L, p = 0.176). The adjusted odds ratio for WBC was 1.05 (95 % CI 1.02–1.08), confirming the robustness of our primary results.

## 4 Discussion

In this large cohort study of aSAH patients, we found significant associations for WBC with the development of DCI, for the combination of WBC with PC and the combination of WBC with established predictors mFS and WFNS. However, we did not find a significant association for PC alone. WBC had better predictive accuracy than established predictors mFS and WFNS. The model combining all variables WBC, PC, WFNS and mFS provided the highest predictive value. Despite these findings, it is important to note that the predictive value of all variables and discrimination of the model was low to moderate.

Given the inherent challenge in diagnosing DCI – where the timing and occurrence remain unpredictable – early risk stratification is of paramount clinical importance. Identifying patients at higher risk using readily available biomarkers, such as WBC and PC, can facilitate targeted surveillance and prompt intervention. In practice, patients deemed to be more prone to developing DCI may benefit from increased monitoring frequency, early diagnostic imaging, and, when necessary, transfer to higher levels of care. These measures could potentially lead to earlier detection and improved management of DCI, ultimately enhancing patient outcomes.

In the last decennia, a paradigm shift has occurred where vasospasm as the sole aetiology of DCI has been replaced by a more multifactorial mechanism. Although the complete pathophysiology of DCI has yet to be elucidated, recent reviews have categorized the mechanism in three broad processes: vascular dysfunction, inflammation, and spreading depolarization (Dodd et al., 2021). Neuroinflammation, characterized by activation of immune cells and release of inflammatory mediators in the central nervous system, has been increasingly recognized as a significant contributor to the risk of DCI in patients suffering from aSAH. Following the rupture of intracranial aneurysms, an inflammatory cascade ensues leading to the release of blood products into the subarachnoid space and subsequent activation of immune responses, perpetuating chronic neuroinflammatory processes. The larger the hemorrhage, the more severe the reaction. This sustained inflammation not only exacerbates neuronal damage but also potentially contributes to the pathogenesis of DCI in aSAH patients. Several studies have highlighted the pivotal role of neuroinflammation in potentiating the risk of DCI post-aSAH, also emphasizing its potential as a therapeutic target (Schneider et al., 2018; Ducruet et al., 2010). Taking this knowledge about neuroinflammation into account, variables representing this phenomenon could mitigate in risk stratification of DCI in this vulnerable patient population. Although WBC and PC are peripheral blood parameters and are not directly related to neuroinflammation in the central nervous system, it is conceivable that these processes are interconnected. Ideally, one would assess parameters that directly reflect the central nervous system, such as those obtained through a lumbar puncture. However, this was beyond the scope of our study, as lumbar punctures are not routinely performed on every patient in our hospital. Our study specifically aimed to identify available parameters at the time of hospital admission as predictors of DCI. Additionally, the variables included in our study have already been associated with DCI in the literature, and we aimed to further explore this association.

In our cohort, mean WBC levels were elevated above the normal range on admission despite most blood samples being obtained within 10 h of ictus. Although leukocytosis and thrombocytosis typically require 24–96 h to develop due to bone marrow production, the acute phase of aSAH is associated with an immediate inflammatory response. This response, driven by mechanisms direct inflammatory signaling, likely accounts for the early elevation in WBC. Thus, our findings support the notion that aSAH induces rapid immune dysregulation, which may serve as an early indicator of disease severity. In addition, our results showed that WBC was significantly higher in the DCI group compared to the group without DCI, indicating that the inflammation process is even more pronounced in these patients. In the acute phase after any type of trauma, blood cells can respond and multiply quickly. Regarding other acute brain injuries, higher WBC has previously been correlated with severe head injury versus minor head injury, and greater degree of impairment, disability and mortality following ischemic stroke (Rovlias and Kotsou, 2001; Furlan et al., 2014).

Studies have shown that physiological parameters start reacting after the ictus of SAH, and have also found that this is associated with the occurrence of DCI. Mcgirth et al. showed that WBC tends to rise in the first days after SAH, peaking at day five, and that peak WBC greater than 15.0 × 10^9^/L increases the odds of vasospasm threefold (McGirt et al., 2003). Mahta et al. showed that WBC reaches a peak at a median (IQR) of two (1.0–2.5) hospital days for patients with good outcome and one (1.0–3.0) hospital day for patients with poor outcome. Additionally, this peak WBC was associated with DCI (Mahta et al., 2021). Ray et al. reported that reactive platelet count tends to rise in the first three days in patients with DCI, as defined by ratio of mean platelet volume to platelet count (MPV:PC) (Ray et al., 2018a). Considering this, it is plausible that laboratory parameters in early hospital admission course can serve as potential predictors, as indicated by our described significant associations. Nevertheless, the fact remains that the predictive value of WBC and PC in our study is low.

### 4.1 Literature in context

Studies on the value of WBC and PC in the prediction of DCI after aSAH show ambiguous results, leaving their value is inconclusive. Below, we provide a brief review of literature.

Some studies have demonstrated significant effect, supporting the inclusion of WBC in prediction models. Hu et al. showed that high WBC combined with high Hounsfield Units (HU) of blood clots in the subarachnoid space resulted in an OR of 36.89 (CI 5.61–242.78) with a high predictive value (AUC 0.90, CI 0.94–0.96). However, when the parameters were evaluated separately, they yielded smaller ORs, comparable to those in our study, although the AUCs were substantially higher (Hu et al., 2022). While these results seem promising, they need validation in studies with larger sample sizes. Al-Mufti et al. reported comparable significant results in a large cohort with similar ORs and AUCs for WBC and other predictors. However, the effect was limited to patients with good clinical admission grade (Al-Mufti et al., 2019b). Besides, they dichotomized WBC at 12.1 × 10^9^/L to denote moderate inflammation - a value is close to the cut-off point for normal ranges of WBC, and in our cohort mean WBC for all patients was already substantially elevated above this cut-off point. Since an inflammatory state is described in all aSAH patients, the clinical usefulness of WBC for predicting DCI with this cut-off point is questionable. Moreover, other studies have presented results of small OR’s and AUCs for WBC in relation to DCI occurrence (Mahta et al., 2021; Chamling et al., 2017). Although the results were just significant, Chamling et al. state that due to the lack of specificity, WBC should not be used as a biomarker. Lastly, several studies have shown that WBC has no predictive value with no significant results in uni- and multivariable analyses (Tao et al., 2017; McMahon et al., 2013; Muroi et al., 2012; Hurth et al., 2020).

The role of platelet count or surrogate parameters is even more debated. Frontera et al. showed that platelet activation was associated with early brain injury (EBI), worse outcome and DCI, but presented a modest OR for DCI of 1.1 (1.0–1.2) (Frontera et al., 2017). Furthermore, Ray et al. indicated trends in that coated-platelet after aSAH predict DCI and short-term clinical outcomes (Ray et al., 2018b). Work-up with these parameters is not routinely performed, and it was thus not feasible to include in the current study, which focused on clinically and routinely useful admission parameters. Furthermore, several studies found no significant association for platelet count on DCI. Chen and Zhang showed that high MPV between 3 and 5 days was associated with development of DCI (OR 4.51, CI 2.68–7.63, AUC 0.83, CI 0.75–0.88), but higher PC between 3 and 5 days had a non-significant OR of 1.004 (0.99–1.02). Previously, this was also demonstrated by Kasius et al., implying that these parameters do tend to rise during the development of DCI (Kasius et al., 2010). However, since onset of DCI might precede these rises, their usefulness as predictors is questionable.

Recently, a study was published by Zhang et al. investigating white blood cell to platelet ratio (WPR) and found a significant association with DCI for WBC, PC and WPR (Zhang et al., 2023). The study focused specifically on WPR as prognostic biomarker for DCI. WPR is a distinct different parameter, since it is a ratio between WBC and PC. Our objective was to specifically assess the combination of WBC and PC. Our goal was to create a model based on readily available variables upon admission, in our opinion it might be too demanding for a clinician to manually calculate the ratio in an acute setting. Furthermore, it is always important to observe similar analysis in different large cohorts bring forth similar results, since data for the Chinese population does not necessarily apply to populations from other regions of the world. The Asian population has different characteristics, take for example the higher incidence of Intracranial Atherosclerotic Disease (ICAD). Overall, the results of Zhang et al. and our results, highlight the importance of the observed parameters and the association with DCI following aSAH.

In our study, the predictive values of WBC exceeded those reported in most existing literature. It appears that the effect of WBC is more pronounced than the effect of PC, which can also be deduced from previous studies. Remarkably, this study has even shown that WBC even had a higher predictive value than the already established and well-known predictors mFS and WFNS or their combination. Despite this, the predictive abilities of all our variables and models are low to moderate, indicating that these variables and models do not sufficiently discriminate for prediction of DCI. This lack of strong predictive power represents a significant limitation. The models, as they currently stand, are not robust enough to be used in clinical practice. They fail to provide a reliable means of predicting DCI, which diminishes their potential utility and relevance for clinicians who rely on accurate models for decision making. While the inclusion of WBC shows some promise by improving the predictive value to a certain extent, this improvement is not substantial enough to overcome the inherent limitations of the models. Therefore, the models in this construction will presumably not influence clinical practice. In future studies when models with better predictive abilities are created, it could be worthwhile to investigate whether adding WBC is of value.

Lastly, we conducted the sensitivity analysis to improve generalizability. In the primary analysis, we excluded patients with DCI and death within 72 h after ictus, to ensure that blood counts preceded the development of DCI and to create a cohort with patients who have the potential to develop DCI. For the sensitivity analyses, we only excluded the patients with DCI before first blood counts. In this larger cohort the results were similar; however, the effect size was smaller for WBC. This reinforces the idea that WBC or PC in this form are not of substantial use in these prediction models.

### 4.2 Strengths & limitations

The principal strengths of this study are the large cohort of 660 included aSAH patients and therefore the representativeness of the population. Additionally, the investigated parameters and predictors together cover a wide range of data, including radiological, clinical and physiological. In addition, the investigated variables are part of routine admission work-up, are easy to use in decision making and are available at large scale, making them appealing as potential predictors.

However, this study has several limitations, the most important one being the weak predictive power of all variables and models. As demonstrated in the results, the discrimination of a prediction model based purely on WBC, PC, mFS and WFNS is poor to moderate. While WBC appears to be an important predictor of DCI in this population future studies are necessary to provide recommendations to practice based on this marker alone. While our analysis shows a modest difference in WBC between patients with and without DCI, the large individual variations and low predictive value highlight the need for caution in using WBC alone for clinical decision-making in DCI risk assessment. Even though the predictive power of WBC is higher than in most literature, and even higher than established predictors, it is doubtful that they are clinically useful. Another limitation is the retrospective nature of the study, although the data of the SAH-registry is prospectively gathered. Furthermore, WBC differentiation is not part of the routine laboratory sampling and therefore we were not able to further specify which type of WBC show the found association with DCI even though this could provide valuable insights. Lastly, given that true DCI within the first 72 h post-rupture is extremely rare, the initial admission bloodwork – obtained at a median time of 4 h post-ictus – likely reflects the pre-DCI state of the patient. Although rare instances of delayed blood sampling may have occurred, particularly during the transition to an electronic patient dossier in 2015, these exceptions are infrequent and do not materially affect our results.

## 5 Conclusions

This study focused on the association of WBC, PC and especially their combination with DCI. Our results show a significant association for WBC with the development of DCI. Among all evaluated variables - qualifying variables WBC and PC and established predictors mFS and WFNS, as well as their combination - WBC demonstrated the strongest predictive value. However, it should be noted that the predictive value of WBC and PC, as well as that of established predictors or the combination, appears low to moderate. This significant limitation suggests that the current models are not robust enough for clinical use. Predicting DCI remains a challenging task and the potential role of biomarkers and big data in prediction of DCI remains an interesting and important topic to be studied. Incorporation of these parameters, and especially WBC, does improve the prediction model. Future research should continue focusing on these types of variables of DCI and try to create more robust models with higher predictive accuracy. Only then would it be meaningful to re-evaluate the contribution of WBC to these enhanced models.

## CRediT authorship contribution statement

**Eva Postma:** Conceptualization, Methodology, Formal analysis, Investigation, Writing – original draft, Writing – review & editing. **Homeyra Labib:** Formal analysis, Investigation, Writing – review & editing. **Jordi van Lange:** Formal analysis, Investigation, Writing – original draft. **Bert Coert:** Writing – review & editing. **René Post:** Writing – review & editing. **René van den Berg:** Funding acquisition, Resources, Supervision. **Charles Majoie:** Funding acquisition, Resources, Supervision. **W. Peter Vandertop:** Conceptualization, Supervision. **Dagmar Verbaan:** Conceptualization, Writing – review & editing, Funding acquisition, Resources, Supervision.

## Informed consent statement

N/A.

## Institutional Review Board statement

Investigations were carried out following the rules of the Declaration of Helsinki of 1975, revised in 2013. Approvals for this retrospective cohort study and the prospective SAH registry were obtained from the Institutional Review Board committee (Medical Ethics Committee AMC, reference number resp. W19_297, 19.354 31/07/2019 and W11_145, 11.17.0979).

## Data availability

The data presented in this study are available on request from the corresponding author due to privacy reasons.

## Funding

No funding was received for conducting this study.

## Declaration of competing interest

The authors declare the following financial interests/personal relationships which may be considered as potential competing interests.
