# Supplementary material for: The value of white blood cell count and platelet count in predicting delayed cerebral ischemia after aneurysmal subarachnoid hemorrhage
Source: Brain Spine. 2025 Mar 13;5:104236. doi: 10.1016/j.bas.2025.104236 (PMC12391828; doi:10.1016/j.bas.2025.104236)

**Supplementary material**

We tested the possibility of effect modification by fitting a model that included all the previously described predictors, along with a multiplicative interaction term between WBC and PC. To assess the significance of the interaction, we performed a log-likelihood ratio test comparing the original model (without the interaction term) to the model with the interaction term. The resulting p-value of 0.43 suggests that an interaction is unlikely. We produced a plot of the predicted probabilities allowing for the possibility of effect modification but as demonstrated in the plots the PC values does not appear to have influence of the association between WBC and the risk of DCI.

1. Plot of the predicted probabilities


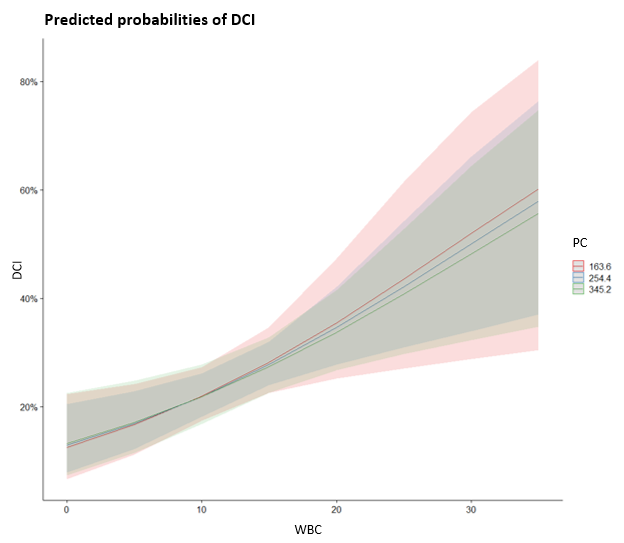

Supplement: Multimedia component 1 [file mmc1.docx]
